# Diagnostic value of quantification of cell‐free DNA for suspected gallbladder cancer

**DOI:** 10.1002/jgh3.12977

**Published:** 2023-09-29

**Authors:** Katsunori Sakamoto, Kohei Ogawa, Kei Tamura, Masahiko Honjo, Kyosei Sogabe, Chihiro Ito, Miku Iwata, Akimasa Sakamoto, Mikiya Shine, Yusuke Nishi, Mio Uraoka, Tomoyuki Nagaoka, Naotake Funamizu, Yasutsugu Takada

**Affiliations:** ^1^ Department of Hepato‐Biliary‐Pancreatic and Breast Surgery Ehime University Graduate School of Medicine Ehime Japan

**Keywords:** carcinoembryonic antigen, cell‐free DNA, gallbladder cancer, tumor depth

## Abstract

**Background and Aim:**

An accurate preoperative diagnosis as the basis for deciding the most appropriate surgical procedure is essential for patients with suspected gallbladder cancer (GBC). The aim of this study was to investigate the usefulness of cell‐free DNA (cfDNA) for the preoperative detection of ≥T2 invasion in patients with suspected GBC.

**Methods:**

Twenty‐four patients who underwent resection for suspected GBC were enrolled. The concentration of cfDNA obtained from blood samples preoperatively was measured and evaluated in two distributions. The first peak (less than 200 base pairs) of cfDNA distribution was defined as the shorter fragment cfDNA, considered to originate mainly from apoptosis; and the second peak (200 base pairs or more) was defined as the longer fragment cfDNA, originating mainly from necrosis.

**Results:**

Pathological analysis identified benign disease in 12 patients and GBC in 12 patients, of whom 6 patients had ≥pT2 GBC. Carcinoembryonic antigen (CEA) and carbohydrate antigen (CA)19‐9 were significantly higher in the ≥pT2 GBC group than in the benign/<pT2 groups (2.1 [0.7–11.0] *vs* 4.5 [1.7–13.0], *P* = 0.033 and 14.0 [<2.0–401] *vs* 37.0 [26.0–141.0], *P* = 0.007, respectively). When limited to patients in the GBC group (*n* = 12), only cfDNA of longer fragments was significantly lower in the ≥pT2 group than the <pT2 groups (2.98 [1.88–4.61] *vs* 1.98 [1.42–2.42], *P* = 0.026) but cfDNA of shorter fragments showed no significant difference between above both comparisons.

**Conclusion:**

CfDNA might have potential use as a diagnostic factor for patients with suspected GBC.

## Graphical abstract

The aim of this study was to investigate the usefulness of cell‐free DNA (cfDNA) value for the preoperative detection of ≥T2 invasion in patients with suspected gallbladder cancer. cfDNA of longer fragments was significantly lower in the ≥pT2 group than in the <pT2 group. CfDNA might have potential use as a diagnostic factor for the patients with suspected gallbladder cancer.

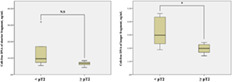

## Introduction

Gallbladder cancer (GBC) is a relatively rare cancer that has high malignant potential and poor prognosis.^1^ Radical resection is the only curative treatment, but as the disease is commonly in an advanced stage by the time of diagnosis, few patients qualify for resection.^1^, ^2^ Although recent advances in imaging modalities have enabled early detection of GBC,^3^ an accurate preoperative diagnosis remains challenging.^3^ Furthermore, in those deemed suitable for resection, the choice of procedure varies according to the degree of progression.^2^ Simple cholecystectomy can be performed for GBC within the submucosal layer, whereas radical resection with combined resection of the gallbladder bed with regional lymphadenectomy is required in the case of invasion to the subserosal connective tissue (T2) and beyond, which is associated with a 40–50% incidence of lymph node metastasis (LNM).^3^, ^4^, ^5^ Thus, the ability to diagnose T2 or more preoperatively is important for patients with suspected GBC. Although recent advances in minimally invasive surgery for GBC can solve this dilemma even for advanced GBC,^6^ this approach has not been accepted worldwide. Therefore, an accurate preoperative diagnosis as the basis for deciding the most appropriate surgical procedure is essential for patients with suspected GBC. Furthermore, Gallbladder wall thickening in chronic cholecystitis or xanthogranulomatous cholecystitis (XGC), which often mimics advanced GBC, is often included in suspected cases of GBC and can confuse the selection of appropriate surgical management. For these reasons, it is important to obtain an accurate diagnosis in patients with suspected GBC, including the extent of tumor progression (distinguishing progression T2 or higher from less than T2 or benign lesions) to enable appropriate management of GBC.^1^, ^3^ Various methods have been reported for assessing the depth of invasion preoperatively^1^, ^3^ but no consensus has been reached so far.

Cell‐free DNA (cfDNA) comprises extracellular nucleic acids found in human serum. Its level can vary with disease progression and may have potential as a prognostic biomarker that is minimally invasive for patients. In a healthy individual, cfDNA originates from the apoptosis of nucleated cells, whereas in cancer patients the origin is generally tumor cells.^7^ Hence, cfDNA levels are useful for differentiating cancer patients from healthy individuals.^8^ Previous studies have reported the detection of higher cfDNA levels in several malignant diseases compared with healthy controls.^9^, ^10^, ^11^, ^12^, ^13^ However, an association between cfDNA level and GBC has been reported only by Kumari et al.^14^, ^15^ In addition, cfDNA is useful for predicting the prognosis as well as an indicator of several malignant diseases.^16^ In other words, cfDNA may be a useful predictor of the clinical stage of a malignant disease. Indeed, Kumari et al. have demonstrated the utility of cfDNA for preoperative diagnosis in advanced GBC.^14^, ^15^ However, few studies have reported the usefulness of cfDNA for obtaining a precise preoperative diagnosis in terms of distinguishing lesions that have progressed to T2 or higher from benign lesions and from those less than T2. Thus, the aim of this study was to investigate the usefulness of cfDNA for the preoperative detection of ≥T2 invasion in patients with suspected GBC.

## Methods

### Patients

This was prospective study initiated since January 2020. Enrolled in the study were 24 patients with suspected GBC who underwent surgical resection between January 2020 and February 2023. None of the patients underwent preoperative chemotherapy.

Those who were diagnosed with suspected GBC with initially resectable condition were included in the study. The definition of suspected GBC was a broad‐based mass including focal or diffuse thickening of the wall, or a tumor ≥1 cm with a tendency to increase in size. Patients with suspected GBC who did not undergo resection due to locally advanced or metastatic disease considered as initially unresectable, or those who were inoperable due to general conditions or co‐morbidity, were excluded. Patients who declined to participate in the present study were also excluded.

We evaluated correlations of preoperative findings such as blood biochemistry data, radiographic findings, and fluorodeoxyglucose‐positron emission tomography (FDG‐PET) with the cfDNA and pathological findings. The depth of tumor invasion was defined using the TNM Classification of Malignant Tumors published by the Union for International Cancer Control (UICC), eighth Edition.^17^

### cfDNA

Blood samples (10 ml) were obtained on the day before surgery in all patients, which were collected in Streck BCT tubes (Streck, Omaha NE), then stored at 4 °C, and processed by centrifugation 2000*g* for 10 min at room temperature. The plasma layer was transferred to a new conical tube without removing the buffy coat and stored at −80 °C.

The concentration of cfDNA was measured at Nihon Gene Research Laboratories Inc. (Sendai, Japan), as follows:

After thawing the frozen plasma at 4 °C, large debris was sedimented by centrifugation (2000*g*, 10 min, 20 °C), and 1.5 ml of the supernatant was collected. High‐speed centrifugation (16 000*g*, 10 min, 20 °C) was performed to completely sediment the debris, and 1 ml of the supernatant was transferred to a new tube.

cfDNA was extracted from 1 ml of the pretreated plasma using the MagMAX Cell‐Free DNA Isolation Kit (Thermo Fisher Scientific) according to the manufacturer's specified protocol. The DNA elution volume was set to 15 μl.

Electrophoresis of DNA extracts was performed using the TapeStation2200 and High Sensitivity D5000 reagent kit (Agilent Technologies), and the DNA content was determined by the TapeStation software.

Regarding the cfDNA fragment distribution, the first peak was defined as the shorter fragment, which reportedly derives mainly from apoptosis, and the second peak was defined as the longer fragment, which originates mainly from necrosis.^18^, ^19^, ^20^ Short‐fragment cfDNA generally refers to that with <200 base pairs (bp).^16^

### Ethical considerations

The institutional review board approved this study (Approval No. 1910008), which was conducted in accordance with the ethical standards established in the Declaration of Helsinki in 1995 (revised, Brazil 2013). Written informed consent was obtained from all patients.

### Statistical analysis

Continuous variables were compared using Mann–Whitney U tests and are presented as medians with ranges. Categorical variables were compared using chi‐squared or Fisher exact tests and are presented as numbers with ratios (%). Statistical significance was defined as *P* <0.05. All data were statistically analyzed using the SPSS statistical software version 24.0 (IBM Corp., Armonk, NY, USA).

## Results

**Figure 1:**
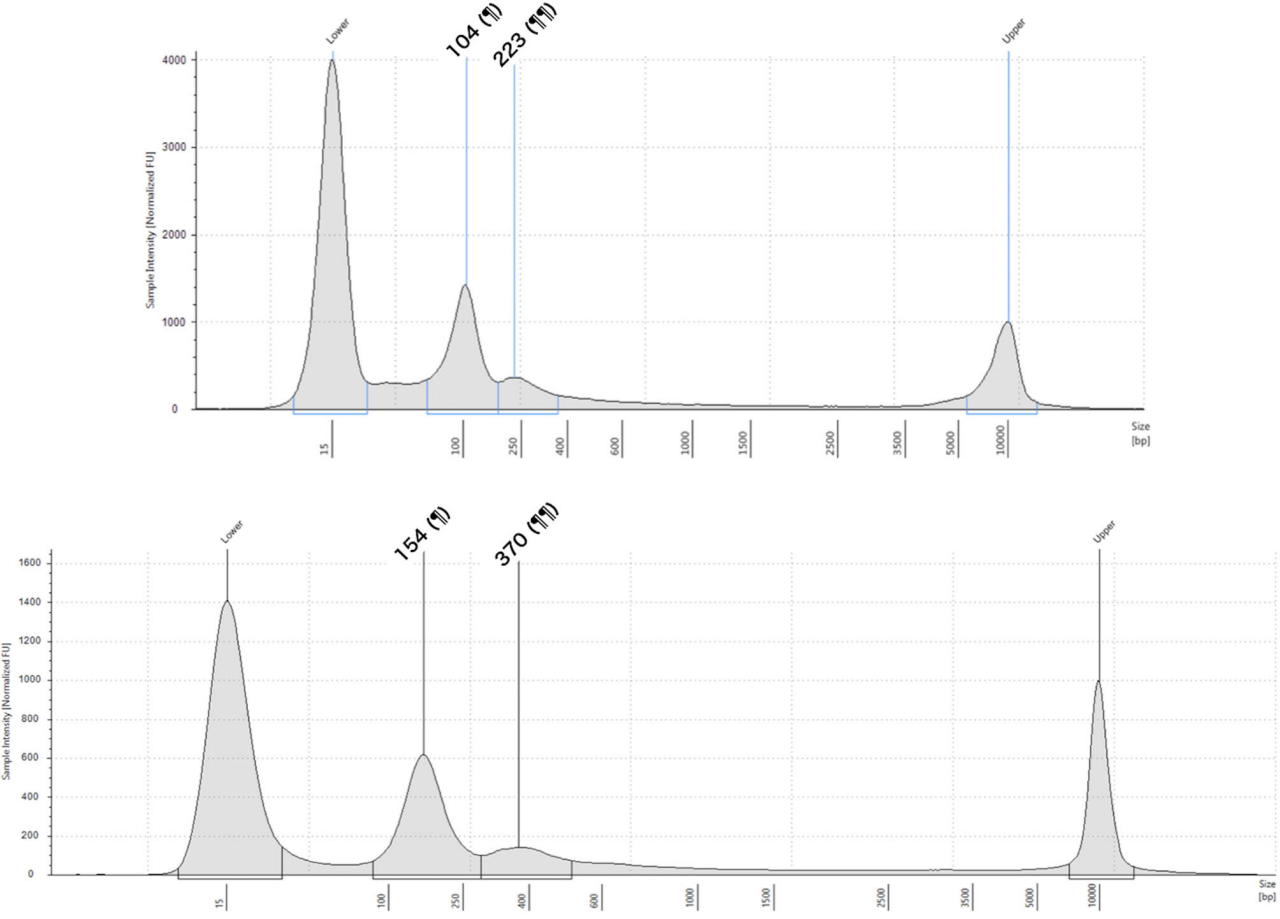
Cell‐free DNA fragment distribution. (a) Representative patient with chronic cholecystitis (case No. 2 in Table 1). (b) Representative patient with gallbladder cancer (case No. 12 in Table 1). ¶ and ¶¶ indicate peak lengths of first (shorter) and second (longer) fragments (base pair), respectively.

Table 1 lists the patient characteristics. Median age was 71 years (range, 43–86 years) and 13/24 patients were male (54.2%). Median BMI was 23.5 kg/m^2^ (range, 16.6–34.8 kg/m^2^). Median carcinoembryonic antigen (CEA), carbohydrate antigen (CA)19‐9, and standard uptake value (SUVmax) in FDG‐PET were 2.7 ng/ml (range, 0.7–13.0), 16 U/ml (<2.0–40.1), and 6.52 (negative to 27.0), respectively. Median length of the shorter fragments was 154 bp (range, 104–165 bp) and that of longer fragments was 333 bp (223–382 bp) (Figure 1a). Median concentration of cfDNA of the shorter fragments was 7.69 ng/ml (4.17–43.65 ng/ml) and that of longer fragments was 2.38 ng/ml (1.36–11.16 ng/ml). The final pathological finding was benign disease in 12 patients (chronic cholecystitis with adenomyomatosis [ADM] in 8, XGC in 2 patients, cholesterol polyp in 1, and adenoma in 1), and GBC in the remaining 12 (T1 gallbladder in 6, T2 GBC without LNM in 2, T2 GBC with LNM in 3, and T3a GBC in 1).

**Table 1:** Patient characteristics

Case No.	Age, (y)	Sex	BMI, kg/m^2^	Final diagnosis	Gall stone	T†	Tumor size, cm	N†	N detail	Histology	Lymphatic invasion‡	Vascular invasion‡	Neural invasion‡	CEA, ng/ml	CA19‐9, U/ml	CRP, mg/dl	SUVmax	Peak length of shorter fragment, bp	Peak length of longer fragment, bp	cfDNA of shorter fragment, ng/ml	cfDNA of longer fragment, ng/ml
1	71	F	17.6	GBC	(+)	1b	8.0	0	0/3	Well	0	0	0	3.4	2.0	0.04	4.3	150	318	7.31	2.33
2	66	M	29.7	Chronic cholecystitis with ADM	(+)	‐	‐	‐	‐	‐	‐	‐	‐	2.7	401.0	0.56	13.5	104	223	34.83	11.16
3	71	M	19.9	Chronic cholecystitis with ADM	(+)	‐	‐	‐	‐	‐	‐	‐	‐	1.8	15.0	0.04	Negative	116	228	12.35	4.40
4	80	M	22.8	GBC	(+)	2	2.0	1	1/10	Poorly	1	0	0	4.7	141.0	0.03	21.5	142	271	5.93	1.68
5	73	M	23.2	XGC	(+)	‐	‐	‐	‐	‐	‐	‐	‐	0.8	13.0	2.92	14.1	118	274	43.65	8.78
6	69	F	28.0	GBC	(+)	2	3.4	0	0/10	Moderate	1	0	1	13.0	65.0	0.05	2.8	157	334	7.16	2.27
7	74	M	26.3	GBC	(+)	1a	8.0	0	0	Well	0	0	0	1.7	18.0	0.14	27.0	163	358	9.50	3.29
8	43	F	17.2	Chronic cholecystitis with ADM	(+)	‐	‐	‐	‐	‐	‐	‐	‐	0.8	16.0	0.03	‐	159	331	7.49	1.67
9	80	F	24.2	GBC	(−)	1b	2.0	0	0	Well	0	0	0	3.3	2.0	0.05	4.7	157	326	9.68	2.66
10	86	F	24.0	GBC	(−)	1b	5.5	0	0	Well	0	0	0	11.0	63.0	0.28	‐	156	321	16.95	4.34
11	78	M	23.0	GBC	(−)	2	2.5	1	1/3	Moderate	1	1	3	8.3	26.0	0.23	6.5	137	323	7.41	2.24
12	58	F	28.7	GBC	(+)	2	8.0	1	3/5	Moderate	1	2	2	1.7	27.0	0.44	9.8	154	370	4.34	1.42
13	75	M	23.8	GBC	(−)	3a	5.5	0	0/7	Moderate	2	0	0	3.0	32.0	0.10	8.4	153	368	8.46	2.42
14	69	F	16.6	Chronic cholecystitis with ADM	(−)	‐	‐	‐	‐	‐	‐	‐	‐	2.2	9.9	0.09	1.9	146	318	10.50	2.45
15	63	F	34.8	GBC	(−)	1b	4.4	0	0	Papillary	0	0	0	2.0	7.7	0.10	16.7	148	316	31.80	4.61
16	47	F	26.4	Adenoma	(−)	‐	‐	‐	‐	‐	‐	‐	‐	0.7	5.4	0.12	4.3	147	365	5.55	2.22
17	68	M	24.9	Cholesterol polyp with chronic cholecystitis	(−)	‐	‐	‐	‐	‐	‐	‐x1	‐	2.7	10.0	0.02	‐	154	361	4.17	1.61
18	77	F	22.4	Chronic cholecystitis with ADM	(+)	‐	‐	‐	‐	‐	‐	‐	‐	2.0	16.0	0.18	5.0	131	294	8.78	2.82
19	74	M	18.6	Chronic cholecystitis	(+)	‐	‐	‐	‐	‐	‐	‐	‐	5.1	2.0	0.03	Negative	158	345	5.84	1.36
20	73	M	29.1	Chronic cholecystitis	(+)	‐	‐	‐	‐	‐	‐	‐	‐	1.3	5.9	1.19	Negative	159	366	7.88	3.00
21	56	M	24.8	Chronic cholecystitis with ADM	(+)	‐	‐	‐	‐	‐	‐	‐	‐	2.8	17.0	0.05	5.8	163	376	4.68	1.46
22	69	F	21.0	GBC	(+)	1b	5.0	0	0	Well	0	0	0	1.5	115.0	0.51	9.2	165	382	5.46	1.88
23	71	M	20.7	XGC	(+)	‐	‐	‐	‐	‐	‐	‐	‐	3.6	15.0	1.50	11.2	160	340	11.63	2.88
24	80	M	21.4	GBC	(−)	2	7.0	0	0/1	Well	0	0	0	4.3	42.0	0.05	8.9	158	351	6.27	1.71

^†^ Described in accordance with the TNM Classification of Malignant Tumors published by the Union for International Cancer Control (UICC), 8th Edition [32].

^‡^ 0, no invasion; 1, minimal invasion; 2, moderate invasion; 3, severe invasion.

T1a, tumor invades lamina propria; T1b, tumor invades muscular layer; T2, tumor invades perimuscular connective tissue without extension beyond serosa or into liver; T3a, tumor perforates the serosa and/or directly invades the liver and/or one other adjacent organ or structure, such as the stomach, duodenum, colon, pancreas, or omentum; N0, no evidence of lymph node metastasis; N1, metastasis in 1–3 lymph nodes.

ADM, adenomyomatosis; BMI, body mass index; CA19‐9, carbohydrate antigen 19–9; CEA, carcinoembryonic antigen; cfDNA, cell‐free DNA; GBC, gallbladder cancer; F, female; M, male; SUVmax, maximum value of standard uptake value in fluorodeoxyglucose‐positron emission tomography; XGC, xanthogranulomatous cholecystitis.

Although no factor showed statistically significant difference between the benign and GBC groups, a tendency of higher CEA and CA19‐9 was observed in the GBC group (2.1 [0.7–5.1] *vs* 3.4 [1.5–13.0], *P* = 0.052; and 14.0 [<2.0–401.0] *vs* 29.5 [<2.0–141.0], *P* = 0.078, respectively) (Table 2). CEA and CA19‐9 were significantly higher in the ≥pT2 GBC group than in the benign/<pT2 groups (2.1 [0.7–11.0] *vs* 4.5 [1.7–13.0], *P* = 0.033 and 14.0 [<2.0–401] *vs* 37.0 [26.0–141.0], *P* = 0.007, respectively) (Table 2). cfDNA concentration of longer fragments was lower in the ≥pT2 GBC group than in the benign/<pT2 group but did not reach statistical significance (2.74 [1.36–11.16] *vs* 1.98 [1.42–2.24], *P* = 0.090). SUVmax showed no significance in either group (Table 2).

**Table 2:** Comparison of benign or malignancy and benign/< pT2 or ≥pT2 in suspected GBC patients.

	Benign, *n* = 12	GBC, *n* = 12	*P* value	Benign/<pT2, *n* = 18	≥pT2, *n* = 6	*P*‐value
Age, years	70 (43–77)	75 (58–86)	0.052	71 (43–86)	77 (58–80)	0.199
Male sex	8 (66.7%)	5 (41.7%)	0.219	9 (50.0%)	4 (66.7%)	0.410
BMI	22.8 (16.6–29.7)	23.9 (17.6–34.8)	0.443	23.6 (16.6–34.8)	23.4 (21.4–28.7)	0.626
Gall stone, yes	9 (75.0%)	6 (50.0%)	0.200	12 (66.7%)	3 (50.0%)	0.397
CEA, ng/ml	2.1 (0.7–5.1)	3.4 (1.5–13.0)	0.052	2.1 (0.7–11.0)	4.5 (1.7–13.0)	0.033
CA19‐9, U/ml	14.0 (<2.0–401.0)	29.5 (<2.0–141.0)	0.078	14.0 (<2.0–401)	37.0 (26.0–141.0)	0.007
CRP, mg/dl	0.11 (0.02–2.92)	0.10 (0.03–0.51)	0.932	0.11 (0.02–2.92)	0.08 (0.03–0.44)	0.673
SUVmax	4.64 (negative–14.10)	8.93 (2.80–27.00)	0.114	4.96 (negative–27.00)	8.65 (2.80–21.45)	0.470
Peak length of shorter fragment, base pair	155 (104–163)	155 (137–165)	0.478	155 (104–165)	154 (137–158)	0.626
cfDNA of shorter fragment, ng/ml	8.33 (4.17–43.65)	7.36 (4.34–31.80)	0.630	9.14 (4.17–43.65)	6.72 (4.34–8.46)	0.119
Peak length of longer fragment, base pair	336 (223–376)	330 (271–382)	0.590	329 (223–382)	343 (271–370)	0.537
cfDNA of longer fragment, ng/ml	2.64 (1.36–11.16)	2.30 (1.42–4.61)	0.799	2.74 (1.36–11.16)	1.98 (1.42–2.24)	0.090

Continuous variables were compared using Mann–Whitney *U* tests and are presented as medians with ranges. Categorical variables compared using chi‐squared or Fisher exact tests are presented as numbers with ratios (%).

BMI, body mass index; CA19‐9, carbohydrate antigen 19–9; CEA, carcinoembryonic antigen; cfDNA, cell‐free DNA; CRP, C‐reactive protein; GBC, gallbladder cancer; SUVmax, maximum value of standard uptake value in fluorodeoxyglucose‐positron emission tomography; T, tumor invasion status.

**Figure 2:**
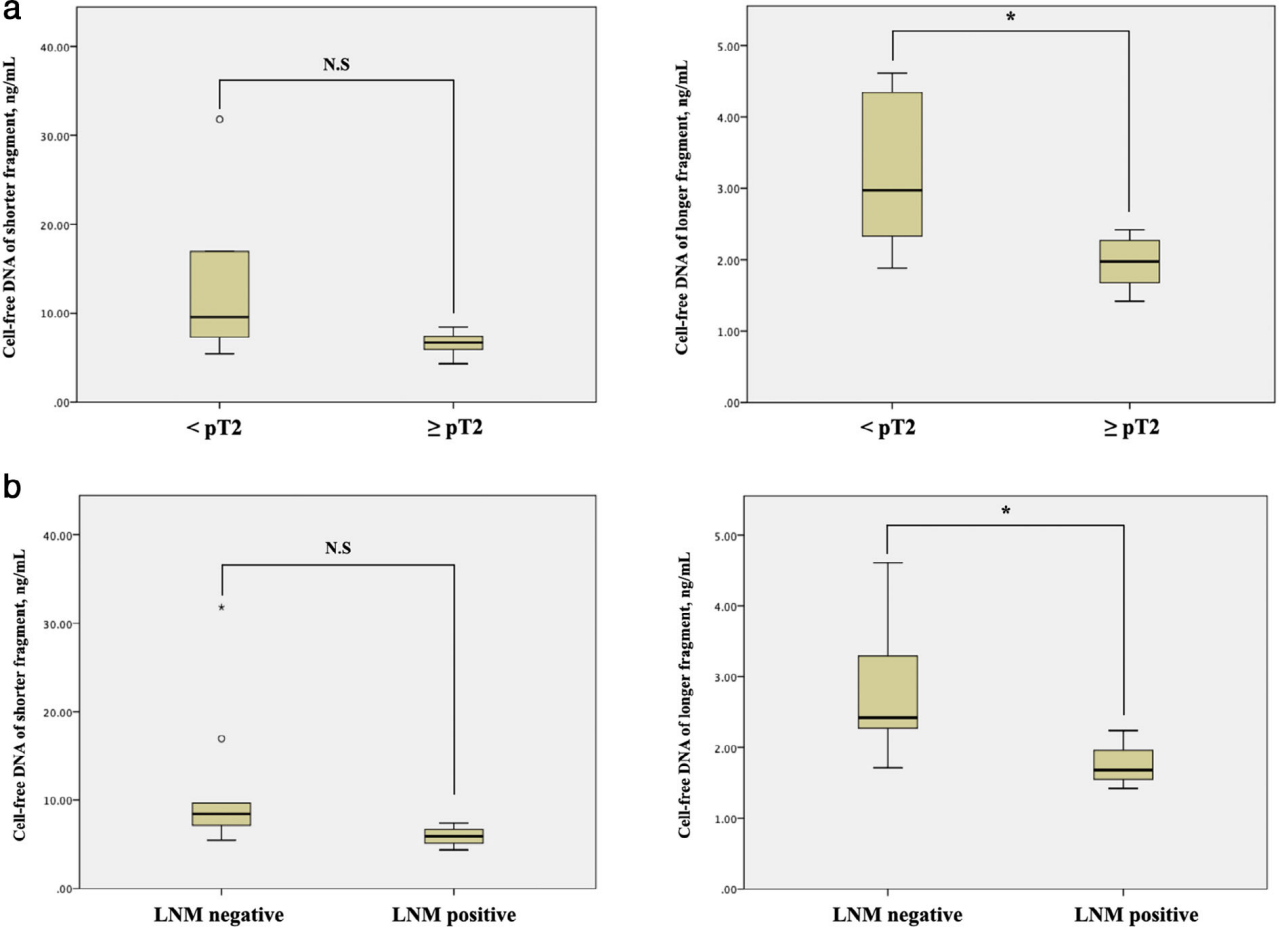
Cell‐free DNA values in patients with gallbladder cancer. (a) Comparison between <pT2 and ≥pT2. Shorter fragment and longer fragment values are shown on the left and right, respectively. (b) Comparison between positive lymph node metastasis and ≥negative lymph node metastasis. Shorter fragment and longer fragment values are shown on the left and right, respectively. LNM, lymph node metastasis; N.S., not significant; * *P* < 0.05 (*P* = 0.026 and *P* = 0.036, respectively).

When limited to patients in the GBC group, only cfDNA of longer fragments was significantly lower in both the ≥pT2 and LNM (+) group than the <pT2 and LNM (−) group (2.98 [1.88–4.61] *vs* 1.98 [1.42–2.42], *P* = 0.026 and 2.42 [1.71–4.61] *vs* 1.68 [1.42–2.24], *P* = 0.036, respectively) but cfDNA of shorter fragments showed no significant difference between the above both comparisons (Table 3 and Figure 2). CEA, CA19‐9, and SUVmax showed no significance in either group.

**Table 3:** Comparison of <pT2 or ≥pT2 and lymph node positive or negative in GBC patients.

	<pT2, *n* = 6	≥pT2, *n* = 6	*P* value	LNM (−), *n* = 9	LNM (+), *n* = 3	*P*‐value
Age, years	73 (63–86)	77 (58–80)	1.000	74 (63–86)	78 (58–80)	1.000
Male sex	1 (16.7%)	4 (66.7%)	0.121	3 (33.3%)	2 (66.7%)	0.364
BMI	24.1 (17.6–34.8)	23.4 (21.4–28.7)	1.000	24.0 (17.6–34.8)	23.0 (22.8–28.7)	1.000
Gall stone, yes	3 (50.0%)	3 (50.0%)	0.716	4 (44.4%)	2 (66.7%)	0.500
CEA, ng/ml	2.7 (1.5–11)	4.5 (1.7–13.0)	0.240	3.3 (1.5–13.0)	4.7 (1.7–8.3)	0.727
CA19‐9, U/ml	12.9 (<2.0–115.0)	37.0 (26.0–141.0)	0.180	32.0 (<2.0–115)	27.0 (26.0–141.0)	0.600
CRP, mg/dl	0.12 (0.04–0.51)	0.08 (0.03–0.44)	0.589	0.10 (0.04–0.51)	0.23 (0.03–0.44)	0.864
SUVmax	9.23 (4.30–27.00)	8.65 (2.8–21.45)	0.792	8.65 (2.8–27.00)	9.8 (6.52–21.45)	0.497
Peak length of shorter fragment, base pair	157 (148–165)	154 (137–158)	0.310	157 (148–165)	142 (137–154)	0.064
cfDNA of shorter fragment, ng/ml	9.59 (5.46–31.80)	6.72 (4.34–8.46)	0.093	8.46 (5.46–31.80)	5.93 (4.34–7.41)	0.145
Peak length of longer fragment, base pair	324 (316–382)	343 (271–370)	0.699	334 (316–382)	323 (271–370)	0.727
cfDNA of longer fragment, ng/ml	2.98 (1.88–4.61)	1.98 (1.42–2.42)	0.026	2.42 (1.71–4.61)	1.68 (1.42–2.24)	0.036

Continuous variables were compared using Mann–Whitney *U* tests and are presented as medians with ranges. Categorical variables compared using chi‐squared or Fisher exact tests are presented as numbers with ratios (%).

BMI, body mass index; CA19‐9, carbohydrate antigen 19–9; CEA, carcinoembryonic antigen; cfDNA, cell‐free DNA; GBC, Gallbladder cancer; LNM, lymph node metastasis; SUVmax, maximum value of standard uptake value in fluorodeoxyglucose‐positron emission tomography; T, tumor invasion status.

Regarding the prognosis of GBC patients, cases 11–13 developed GBC recurrence on postoperative months 5, 3, and 13, respectively. cfDNA concentration in the longer fragment was low in these patients (range, 1.42–2.42) (Table 1).

## Discussion

The present study revealed that cfDNA concentration cannot be used to identify pT2 or higher invasion among patients with suspected GBC, which was the primary endpoint of the present study, but might be useful for identifying pT2 or higher and LNM among GBC patients. Although the usefulness of evaluation by cfDNA was previously reported in GBC,^14^, ^15^ there were no reports that aimed to identify pT2 or higher among suspected GBC patients. Tumor‐related cfDNA has been reported as a candidate prognosticator and biomarker for the detection of several malignant tumors.^21^ Generally, a high concentration of short‐fragment cfDNA indicates advanced malignancy and poor prognosis in cancer patients.^16^ However, the early detection of malignant diseases remains difficult even using circulating tumor DNAs.^22^ In colorectal, gastroesophageal, pancreatic, and breast cancer, the frequency of detectable circulating tumor DNA at Stage I or II was less than 60%.^22^ In the present study, the range of variation in both the shorter and larger fragment groups was too wide to confirm statistical significance while comparing GBC and benign samples. However, the present results suggest that when limited to patients with GBC, quantification of cfDNA concentration might be a useful predictor of advanced stage of the disease.

Regarding the evaluation of cfDNA, for simplicity we quantified only the cfDNA value and did not evaluate gene mutations, which is too costly and difficult to adopt in the clinical setting.^14^, ^15^ If cfDNA were shown to be significant in preoperative diagnosis, it could be easily adopted.

Although the present study showed a significant difference in cfDNA concentration between ≥pT2 and LNM (+) and other groups in GBC patients, the results were not what we expected. Generally speaking, we expected that the cfDNA level would be higher in GBC patients than in those with benign disease.^14^, ^15^ However, concentration of the longer fragment cfDNA was significantly lower in the ≥pT2 and LNM (+) groups than in other groups of GBC patients. We consider that the results disagree because previous evaluations of cfDNA in GBC patients included more advanced tumors, whereas the present cohort included patients only with relatively early stage. Therefore, the diagnostic value of cfDNA might differ in the patient cohort such as relatively early GBC like in the present study.

Another possible reason for this paradoxical result is that the present cohort included only patients with suspected GBC, and no healthy persons were included. The majority of the patients with no malignancy in the present cohort had their diagnosis as cholecystitis. Although the presence of gall stones and the preoperative C‐reactive protein value showed no significance in any of the present patient groups, values of longer fragment cfDNA might be higher in <pT2 and LNM (−) GBC due to necrosis by inflammatory reaction.^23^ The cfDNA value might reflect small differences in inflammation between patients with <pT2 and LNM (−) GBC. Furthermore, a previous study had reported that circulating tumor DNA values were significantly lower in patients with lung‐only or peritoneum‐only colorectal metastases than in those with liver‐only metastases; in other words, the diagnostic power of cfDNA value may vary according to the origin and metastatic site of the tumor.^24^ Other than those hypotheses, it is possible that the diagnostic value of cfDNA evaluation for precise preoperative diagnosis in patients with suspected GBC is not significant. Nevertheless, evaluation of cfDNA or circulating tumor DNA for tumor discrimination in patients with suspected GBC requires further study.

The present study has some limitations. It was conducted with a small number of patients of a single ethnicity. Although we narrowed down the target cases to patients with suspected GBC, it was not enough to demonstrate statistical significance. Even though we could show the significance of cfDNA in such a small number of patients, a larger scale study is warranted. Furthermore, cfDNA size alone cannot be considered a precise indicator for advanced cancer or a diagnostic marker. Therefore, the present observations should be verified by microarray analysis, with hybridizing of the two categories of fragments separately to an array of cancer‐specific target genes, or by using a polymerase chain reaction‐based analysis. However, no previous report has compared cfDNA levels among patients with suspected GBC. A further study may reveal the usefulness of cfDNAs for preoperative diagnosis in these patients and contribute to selecting the most appropriate surgical procedure.

In conclusion, the concentration of longer cfDNA fragments was significantly lower in patients with ≥pT2 or LNM (+) than in patients with GBC without these attributes, and it might thus have potential use as a diagnostic factor. Further study in a larger number of patients is required.
